# Supplementary material for: Bioconversion of CO to formate by artificially designed carbon monoxide:formate oxidoreductase in hyperthermophilic archaea
Source: Commun Biol. 2022 Jun 3;5:539. doi: 10.1038/s42003-022-03513-7 (PMC9166738; doi:10.1038/s42003-022-03513-7)
Supplement: Supplementary file 2 — Supplementary information [file 42003_2022_3513_MOESM2_ESM.pdf]

Supplementary information for

Bioconversion of CO to formate by artificially designed carbon monoxide:formate oxidoreductase in hyperthermophilic archaea

Table of Contents:

Supplementary Tables (Tables 1-6) pages 2-9
Supplementary Figures (Figs. 1-8) pages 10-18
Supplementary Reference pages 19

Supplementary Table 1. Strains, plasmids and fosmids used this study.

Strains/Plasmids/Fosmids	Description	Reference
**Strains**		
E. coli		
EPI300^{TM}-T1^{R}	Fosmid cloning host	EPICENTRE
T. onnurineus		
NA1	Wild-type strain	[1]
D02	NA1 derivative, *Δfdh1* gene cluster (TON_0266-TON_0282) *Δfdh2* gene cluster (TON_1563-TON_1580) *Δfdh3A* (TON_0539)	Previous construction (not reported)
D04	D02 derivative, *Δfdh1* gene cluster (TON_0266-TON_0282) *Δfdh2* gene cluster (TON_1563-TON_1580) *Δfdh3* gene cluster (TON_0539-TON_0541)	This study
D05	D04 derivative pFd3StrepCodhC2008 transformation, P_{0157}*hmgpfu*::*focA-fdh3AB-codhABCD*; Strep-tag inserted in N-terminus of Fdh3A; control strain for the strain BCF13	This study
D06	D04 derivative, *Δcodh* gene cluster (TON_1017-TON_1031)	This study
D07	D06 derivative pCodhN-strepA1142 transformation, P_{0157}*hmgpfu*::*codhABCD*; Strep-tag inserted in N-terminus of CodhB	This study
BCF01	D02 derivative pFd3CoL1C1118 transformation, P_{0157}*hmg$_{pfu}$*::*focA-fdh3ABC*:*codhABCD*; fusion of *fdh3C* and *codhA* with linker $(GGGGS)_1$	This study
BCF02	D02 derivative pFd3CoL2C1119 transformation, P_{0157}*hmg$_{pfu}$*::*focA-fdh3ABC*:*codhABCD*; fusion of *fdh3C* and *codhA* with linker $(GGGGS)_2$	This study
BCF03	D02 derivative pFd3CoL3C1120 transformation, P_{0157}*hmg$_{pfu}$*::*focA-fdh3ABC*:*codhABCD*; fusion of *fdh3C* and *codhA* with linker $(GGGGS)_3$	This study
BCF12	D04 derivative pFd3NHisCoL1C1132 transformation, P_{0157}*hmgpfu*::*focA-fdh3AB*:*codhABCD*; fusion of *fdh3B* and *codhA* with linker $(GGGGS)_1$; His-tag inserted in N-terminus of Fdh3A	This study
BCF13	D04 derivative pFd3NStrepHisCoL1C1149 transformation, P_{0157}*hmgpfu*::*focA-fdh3AB*:*codhABCD*; fusion of *fdh3B* and *codhA* with linker $(GGGGS)_1$; Strep-tag inserted in N-terminus of Fdh3A	This study
**Plasmids**		
pUC118	Backbone plasmid; Amp^{r}	TAKARA
pFdh1,2,3clusterA1135	pUC118 carrying P_{gdh} promotor, HMG cassette, and 1kbp Left-arm (LA) and Right-arm (RA) for deletion of fdh3 gene cluster (TON_0539-TON_0541)	This study

(continued)

Strains/Plasmids/Fosmids	Description	Reference
**Fosmids**		
pCC1FOS	Backbone fosmid; Cm^r	EPICENTRE
pNA1comFosC1096	pCC1FOS carrying P_{0157} promotor, HMG cassette, and 1kbp Left-arm (LA) and Right-arm (RA) for homologous recombination of *T. onnurineus* NA1 genome; backbone fosmid for mutant construction; Sim^r	This study
pFd3CoL1C1118	pNA1comFosC1096 carrying *fdh3* region (*focA-fdh3ABC*) and *codh* region (*codhABCD*) from *T. onnurineus* NA1; fusion of *fdh3C* and *codhA* with linker $(GGGGS)_1$	This study
pFd3CoL2C1119	pFd3CoL1C1118 carrying fusion of *fdh3C* and *codhA* with linker $(GGGGS)_2$	This study
pFd3CoL3C1120	pFd3CoL1C1118 carrying fusion of *fdh3C* and *codhA* with linker $(GGGGS)_3$	This study
pFd3NHisCoL1C1132	pNA1comFosC1096 carrying *fdh3* region (*focA-fdh3AB*) and *codh* region (*codhABCD*) from *T. onnurineus* NA1; fusion of *fdh3B* and *codhA* with linker $(GGGGS)_1$; His_6-tag inserted in N-terminus of Fdh3A	This study
pFd3NHisStrepCoL1C1149	pNA1comFosC1096 carrying *fdh3* region (*focA-fdh3AB*) and *codh* region (*codhABCD*) from *T. onnurineus* NA1; fusion of *fdh3B* and *codhA* with linker $(GGGGS)_1$; pFd3NHisCoL1C1132 carrying Strep-tag inserted in N-terminus of Fdh3A (TON_0539)	This study
pFd3NStrepCodhC2008	pNA1comFosC1096 carrying *fdh3* region (*focA-fdh3AB*) and *codh* region (*codhABCD*) from *T. onnurineus* NA1 without fusion linker; Strep-tag inserted in N-terminus of Fdh3A (TON_0539)	This study
pCodhN-strepA1142	pNA1comFosC1096 carrying *codh* region (*codhABCD*) from *T. onnurineus* NA1; Strep-tag inserted in N-terminus of CodhB (TON_1018)	This study

Supplementary Table 2. PCR primers used in this study. Bold and underlined nucleotides indicate (GGGGS)n linker and tagging sequences, respectively.

Construction/PCR	Nucleotide sequence (5' to 3')	
	**Forward**	**Reverse**
**pNA1comFosC1096**		
Left-arm-P_{0157}-*hmg*	AGCTCGGTACCCGGGGATCCCCAGACCCTCCTCGAGACGA	TGACGCCACGCATGCCTAGGTCATCTCCCAAGCATTTTATGAG
Right-arm	TGCTTGGGAGATGACCTAGGCATGCGTGGCGTCAACGATG	AGGTCGACTCTAGAGGATCCAATGGAATAACCCATCTCACGG
**pFdh1,2,3clusterA1135**		
Left-arm	AATTCGAGCTCGGTACCCGGGGATCCATGGCACAGAATAATTCACTCG	GGCCATCCTTAACAGCACCACCGCCCTATCTTC
Right-arm	GATAGGGCGGTGGTGCTGTTAAGGATGGCCTGTATG	CCAGTGCCAAGCTTGCATGCCTGCAGGAATGACCTTGGTTATGCCG
**pFd3CoL1C1118**		
focA-fdh3C:codhA(GGGGS)$_1$	TAAAATGCTTGGGAGATGACCTAGGATGGCACAGAATAATTCACTCG	GGCAT**GCTGCCTCCGCCGCC**CCCCAGGTAAGCCTCATATTTG
fdh3C:codhA(GGGGS)$_1$*-codhD*	TGGGG**GGCGGCGGAGGCAGC**ATGCCAGCTTTTTCCGGTTC	TGGCCATCGTTGACGCCACGCATGCGACGTCTCACCTCCTGAGTTTAAACCTCAT
**pFd3CoL2C1119**		
focA-fdh3C:codhA(GGGGS)$_2$	TAAAATGCTTGGGAGATGACCTAGGATGGCACAGAATAATTCACTCG	**GCTGCCTCCGCCGCCGCTTCCGCCTCCTCC**CCCCAGGTAAGCCTCATATTTG
fdh3C:codhA(GGGGS)$_2$*-codhD*	**GGCGGCGGAGGCAGCGGAGGAGGCGGAAGC**ATGCCAGCTTTTTCCGGTTC	TGGCCATCGTTGACGCCACGCATGCGACGTCTCACCTCCTGAGTTTAAACCTCAT
**pFd3NHisCoL1C1132**		
focA-His:fdh3A	TAAAATGCTTGGGAGATGACCTAGGATGGCACAGAATAATTCACTCG	TCCTCGTGATGGTGGTGATGGTGCATCCGCACCACCGCCCT
His:fdh3A-fdh3B:codhA(GGGGS)$_1$	GGATGCACCATCACCACCATCACGAGGAGTTTAAGATTGGCCTG	GCTGGGCTGCCTCCGCCGCCCCCGAAGTAGGCGAGCG
fdh3B:codhA(GGGGS)$_1$*-codhD*	TCGGGGGCGGCGGAGGCAGCCCAGCTTTTTCCGGTTCC	TGGCCATCGTTGACGCCACGCATGCGACGTCTCACCTCCTGAGTTTAAACCTCAT

(continued)

Construction/PCR	Nucleotide sequence (5' to 3')	
	**Forward**	**Reverse**
**pFd3NStrepCoL1C1149**		
focA- Strep:fdh3A	TAAAATGCTTGGGAGATGACCTAGGATGGCACAGAATAATTCACTCG	TCCTCCTTCTCGAACTGCGGGTGGGACCACATCCGCACCACCGCCCT
Strep:fdh3A-codhD	GGATGTGGTCCCACCCGCAGTTCGAGAAGGAGGAGTTTAAGATTGGCCTG	TGGCCATCGTTGACGCCACGCATGCGACGTCTCACCTCCTGAGTTTAAACCTCAT
**pFd3NStrepCodhC2008**		
focA-fdh3B	TAAAATGCTTGGGAGATGACCTAGGATGGCACAGAATAATTCACTCG	TGTTGATCACCTCGTTCACCCGAAGTAGGCGAGCG
codhA-codhD	GCCTACTTCGGGTGAACGAGGTGATCAACAATGCC	TGGCCATCGTTGACGCCACGCATGCGACGTCTCACCTCCTGAGTTTAAACCTCAT
**pCodhN-strepA1142**		
codhA	TAAAATGCTTGGGAGATGACCTAGGATGCCAGCTTTTTCCGGTTC	GCGGGTGGGACCACATTTTCACCACCTCAATACC
Strep:codhB-codhD	GGTGGTGAAAATGTGGTCCCACCCGCAGTTCGAGAAGGCCGGAAAGAAGGTTCCC	TGAGGCTGGCCATCGTTGACGCCACGTCGACTCACCTCCTGAGTTTAAACCTC

Supplementary Table 3. Information of the proteins used in this study.

Proteins	Locug tag	M. W. (Da)	NCBI annotation
**Fdh3**			
FocA	TON_0538	35,570	Hypothetical formate transporter
Fdh3A	TON_0539	75,626	Hypothetical formate dehydrogenase, alpha subunit
Fdh3B	TON_0540	18,342	Oxidoreductase iron-sulfur protein
Fdh3C	TON_0541	13,925	4Fe-4S cluster-binding protein
**Codh**			
CodhA	TON_1017	23,581	4Fe-4S ferredoxin, iron-sulfur binding domain protein
CodhB	TON_1018	67,671	carbon-monoxide dehydrogenase, catalytic subunit
CodhC	TON_1019	29,274	Hypothetical ATP-binding protein
CodhD	TON_1020	7,678	Hypothetical RNA-binding protein

Supplementary Table 4. LC-MS/MS identification of purified CFOR subunits showing number of peptides matched and peptide sequence coverage.

No.	Subunit	Accession Number	NCBI Annotation	Predicted M.W. (Da)	Sequence coverage (%)	MASCOT Score
1	Fdh3A	gi|212008644	Hypothetical formate dehydrogenase, alpha subunit	76324	74	2602
2	CodhB	gi|212009124	Carbon-monoxide dehydrogenase, catalytic subunit	68715	79	2704
3	Fdh3B	gi|212008645	Oxidoreductase iron-sulfur protein	19243	81	844
3	CodhA	gi|212009123	4Fe-4S ferredoxin, iron-sulfur binding domain protein	24479	77	1141

Supplementary Table 5. Predicted subunit molar ratio in the CFOR complex.

Subunit	M. W. (kDa)	Predicted number of subunits in the CFOR	Subunit molar ratio	Subunit mass in 50 ug CFOR (ug)	Subunit mole number in 50 ug CFOR (pmol)
Fdh3A	75.6	2	1.8	15.0	198.4
CodhB	67.7	4	3.2	26.7	394.6
Fdh3B-CodhA fusion protein	41.9	2	1	8.3	198.1
CodhA	23.6	2	0.6	5.0	211.9
Fdh3B	18.3	2	0.4	3.3	180.3

Supplementary Table 6. Relative band intensity ratio of FdhA and CodhB from the SDS-PAGE result, Fig 3a. The band intensity was determined by the software Chemidoc MP Image Lab. Fdh3A and CodhB in the CFOR were used as a relative standard individually.

**Subunit**	**Relative band intensity ratio**			
	Lane 1<br>CFOR (6 ug)	Lane 2<br>Fdh3AB (2.2 ug)	Lane 3<br>CodhAB (3.8 ug)	Lane 4<br>Fdh3AB (2.2 ug)<br>+ CodhAB (3.8 ug)
Fdh3A	1	0.96	-	0.92
CodhB	1	-	0.91	0.98

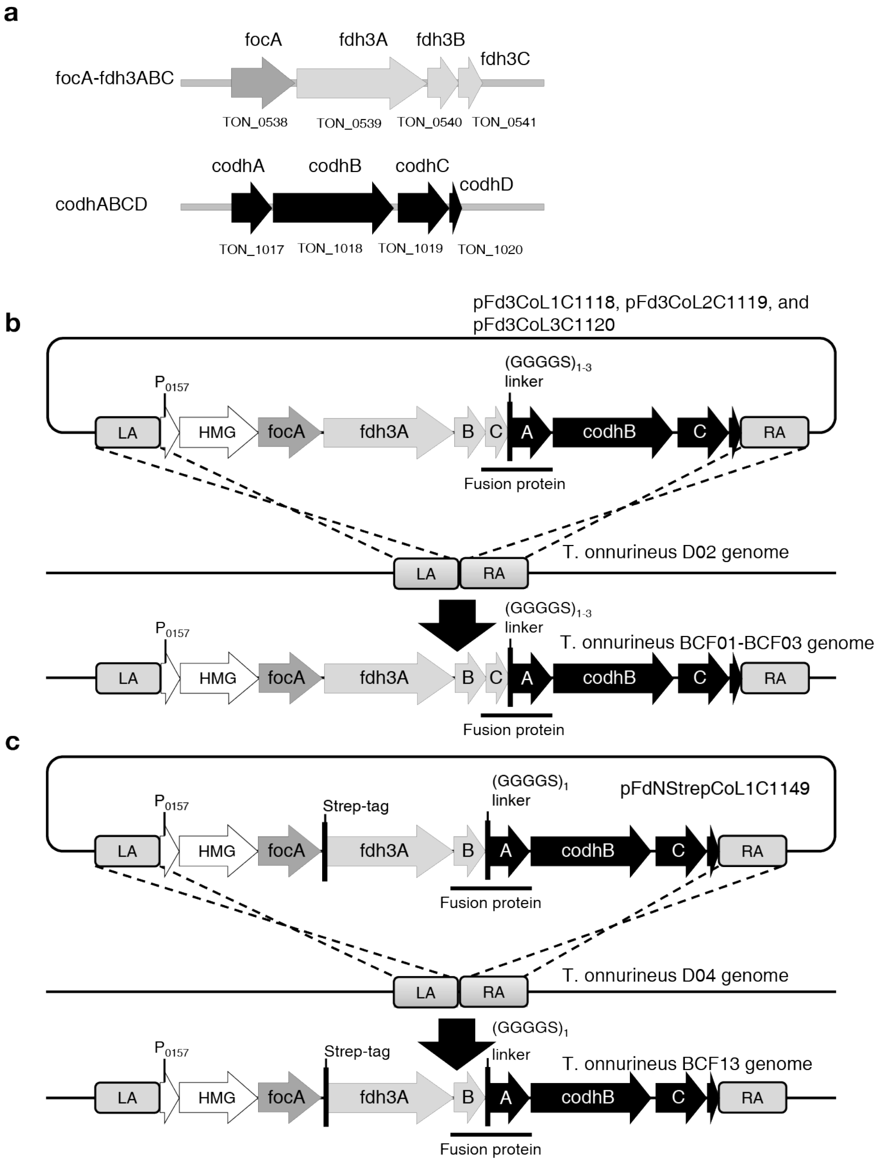

Supplementary Figure 1. Construction of the CO-formate bioconversion mutants by cloning of the synthetic CFOR. a Gene arrangement of the *fdh3* and *codh* gene clusters on the genome of *T. onnurineus* NA1. The name of genes and locus tags are indicated on the upper and lower side of the genes, respectively. **b** Construction of *T. onnurineus* BCF01, BCF02, and BCF03 mutants by transformation and homologous recombination of the *fdh3C:codhA* fusion containing fosmids pFd3CoL1C1118 $(GGGGS)_1$, pFd3CoL1C1119 $(GGGGS)_2$, and pFd3CoL1C1120 $(GGGGS)_3$, respectively. **c** Construction of *T. onnurineus* BCF13 mutant by the transformation of the fosmid pFd3NStrepCoL1C1149, which contains a fusion of *fdh3B:codhA* with linker $(GGGGS)_1$ and strep-tag at the N-terminus of Fdh3A.

a

```
FdnH_Ec       MAMETQDIIKRSATNSITPPSQVRDYKAEVAKLIDVSTCIGCKACQVACSEWNDIRDEVG   60
Fdh3B_To      ---------------------------MEKKLFINLGRCIACRACEVACEKEHGISFIT-   32
CodhA_To      ------------------MPAFSGSNMEKLTIYINPERCTGCRACEIACAVEHSMSKNLF   42
                                          :    *:   * .*:**::**   :.:

FdnH_Ec       HCVGVYDNPADLSAKSWTVMRFSETEQNGKLEWLIRKDGCMHCEDPGCLKACPSAGAIIQ   120
Fdh3B_To      ---------------------------VYEFRDIAVPLNCRHCEKAPCIEVCPT-KAIYR   64
CodhA_To      G--AIFEKPTPKPRL-----------QVVVADFFNVPMRCQHCEDAPCMEACPT-GAISR   88
                                               :     * ***.  *::.**:  ** :

FdnH_Ec       YANGIVDFQSENCIGCGYCIAGCPFNIPRLNKEDNRVYKCTLCVDRVSVGQEPACVKTCP   180
Fdh3B_To      DEDGAVVIDESKCIGCYMCSAVCPYAIPIVDPIKELAVKCDLCAERRKEGRDPLCAAVCP   124
CodhA_To      TKEGFVVLNANKCIGCLMCVMACPFGHPKFEPEYKAVIKCDSCVDRVREGKEPACVEACP   148
                :* * :: .:****  *   **:  * .:   : . **  *.:*   *::* *. .**

FdnH_Ec       TGAIHFGTKKEMLELAEQRVAKLKARGYEHAGVYNPEGVGGTHVMYVLHHA-DQPELYHG   239
Fdh3B_To      TDAIIYADLNELMEEKRRRKAERIVEAQRKA-------VETLAYFG--------------   163
CodhA_To      TRALKFGTLGEILEEVRKEKAESLISGLKSQ---------GMVYMKPVSESKKKEDL--V   197
              * *: :.   *::*  .:. *:    . .               :

FdnH_Ec       LPKDPKIDTSVSLWKGALKPLAAAGFIATFAGLIFHYIGIGPNKEVDDDEEDHHE   294
Fdh3B_To      -------------------------------------------------------   163
CodhA_To      RPMDLYLAYSNVVWY----------------------------------------   212
```

b

```
Fdh3C_To      -VLKVELCVGCGVCAKACPHSAISVFEDSVRRIVFDPKKCEECS-----FECNEACPTGA   54
Fd_Ca         AYVINEACISCGACEPECPVNAISSGD---DRYVIDADTCIDCG------ACAGVCPVDA   51
Fd_Ga         AYVINEACISCGACEPECPVDAISQGG---SRYVIDADTCIDCG------ACAGVCPVDA   51
Fd_Cp         AYKIADSCVSCGACASECPVNAISQGD---SIFVIDADTCIDCG------NCANVCPVGA   51
Fd_Ta         ALYINDDCTACDACVEECPNEAITPGD---PIYVIDPTKCSECVGAFDEPQCRLVCPADC   57
Fd_Av         ALMITDECINCDVCEPECPNGAISQGD---ETYVIEPSLCTECVGHYETSQCVEVCPVDC   57
Fd_Pa         SLKITDDCINCDVCEPECPNGAISQGE---EIYVIDPNLCTECVGHYDEPQCQQVCPVDC   57
                   :  *  *..*   **  .*          .::   * :*        *  .**...

Fdh3C_To      LEGKSDKRELVF--EFAYCAICGKRLNIVKEEAEYLAKKLIELGENPEIAFLCDDCKRKR   112
Fd_Ca         PVQA--------------------------------------------------------   55
Fd_Ga         PVQA--------------------------------------------------------   55
Fd_Cp         PVQE--------------------------------------------------------   55
Fd_Ta         IPDNPDYRETREELQEKYDRLHG-------------------------------------   80
Fd_Av         IIKDPSHEETEDELRAKYERITGEG-----------------------------------   82
Fd_Pa         IPLDDANVESKDQLMEKYRKITGKA-----------------------------------   82

Fdh3C_To      LFGVANKYEAYLG     125
Fd_Ca         -------------     55
Fd_Ga         -------------     55
Fd_Cp         -------------     55
Fd_Ta         -------------     80
Fd_Av         -------------     82
Fd_Pa         -------------     82
```

Supplementary Figure 2. Alignment of the amino acid sequence of Fe-S proteins within the CFOR in *T. onnurienus* (*To*) and other Fe-S proteins in which crystal structure has been determined. The conserved [4Fe-4S] cluster is boxed, and the position of conserved Cys residues are indicated as bold. **a** Amino acid sequences of Fdh3B and CodhA subunits are aligned with 4[4Fe-4S] clusters containing FdnH (PDB 1FDI) in *E. coli* (*Ec*). **b** Multiple

alignments of Fdh3C subunit was achieved with other 2[4Fe-4S] clusters containing ferredoxins (Fd) in *Clostridium acidurici* (*Ca*, PDB 1FDN), *Gottschalkia acidurici* (*Ga*, PDB 1FCA), *Clostridium pasteurianum* (*Cp*, PDB 1CLF), *Thauera aromatica* K172 (*Ta*, PDB 1RGV), *Allochromatium vinosum* (*Av*, PDB 1BLU), and *Pseudomonas aeruginosa* (*Pa*, PDB 2FGO). The multiple alignments were obtained using Clustal Omega program on the webserver (https://www.ebi.ac.uk/Tools/msa/clustalo/).

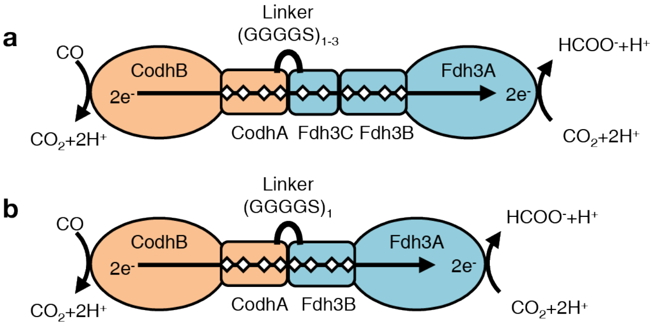

Supplementary Figure 3. Proposed models of synthetic carbon monoxide:formate oxidoreductase (CFOR). a The fusion constructions of Fdh3C-CodhA with linker (GGGGS)$_{1-3}$. **b** The fusion constructions of Fdh3B-CodhA with linker (GGGGS)$_1$.

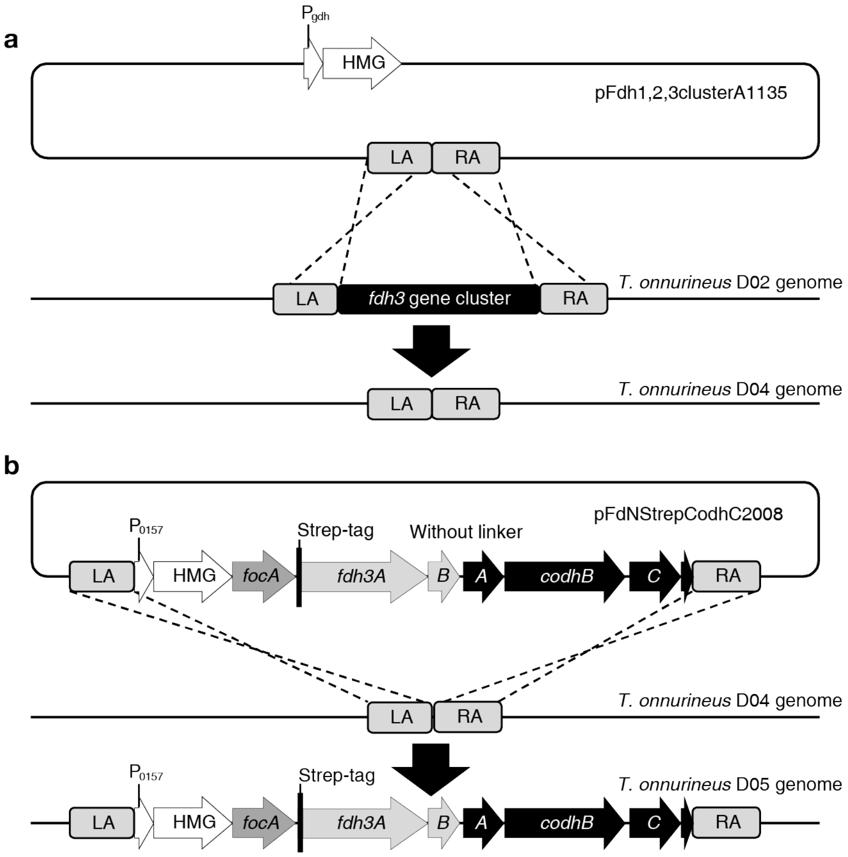

Supplementary Figure 4. Schematic diagram of mutant construction. a Additional deletion of *fdh3* gene cluster for the construction of *T. onnurineus* D04 strain. Strain D02 was transformed with the pFdh1,2,3clusterA1135 vector containing a *fdh3* gene cluster flanked by two ~1 kb DNA fragments, LA (left-arm) and RA (right-arm). Target gene cluster deletion was achieved by double-crossover event. **b** Construction of *T. onnurineus* D05 mutant by the transformation of pFdNStrepCodhC2008 which has not including flexible linker fusion.

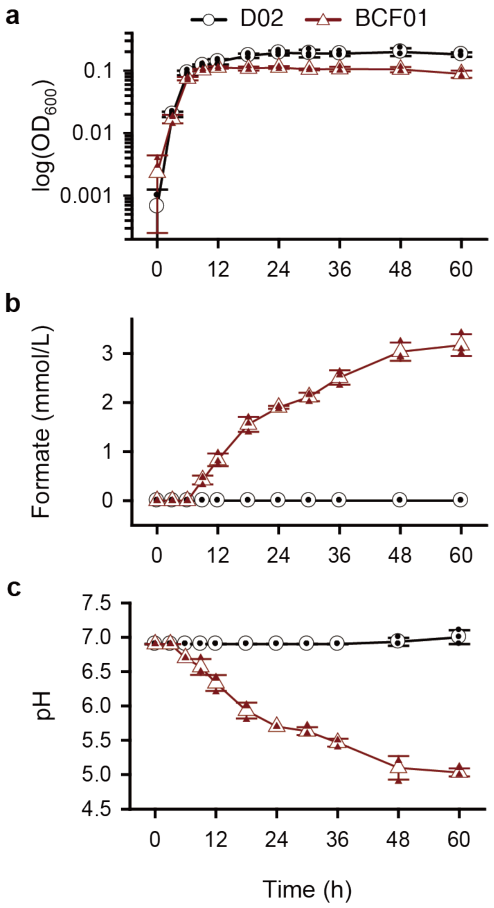

Supplementary Figure 5. Cell growth (a), formate production (b), and pH changes (c) of strain D02 and BCF01 in 100% CO supplemented serum vials. The data were presented as the average ± SD (open symbol), and all the individual data points were shown (closed symbol). Error bars represent ± SD (n = 3).

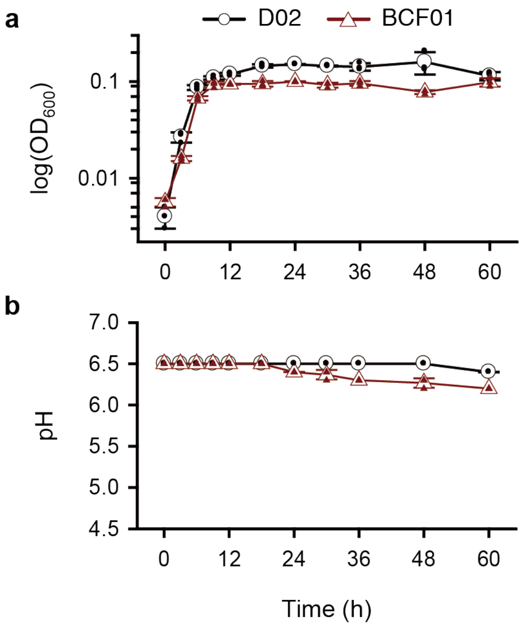

Supplementary Figure 6. Cell growth (a) and pH changes (b) of *T. onnurineus* parental strain D02 and *T. onnurineus* BCF01, with 0.1 M bis-Tris propane buffer (pH 6.5). The data were presented as the average ± SD (open symbol), and all the individual data points were shown (closed symbol). Error bars represent ± SD (n = 3).

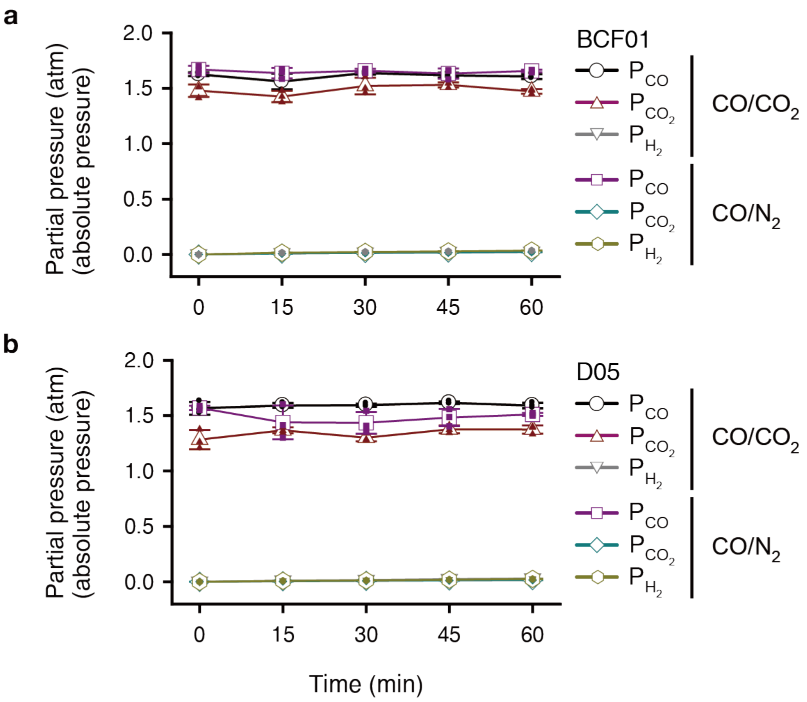

Supplementary Figure 7. Partial pressure changes of *T. onnurineus* BCF 13 (a) and *T. onnurineus* D05 (b) during cell suspension experiments. The headspace was filled with 2 bar (gauge pressure) of CO/CO_2 (50:50 v/v) mix gas or CO/N_2 (50:50 v/v) mix gas. The partial pressure was determined by gas chromatography. The data were presented as the average ± SD (open symbol), and all the individual data points were shown (closed symbol). Error bars represent ± SD (n = 3).

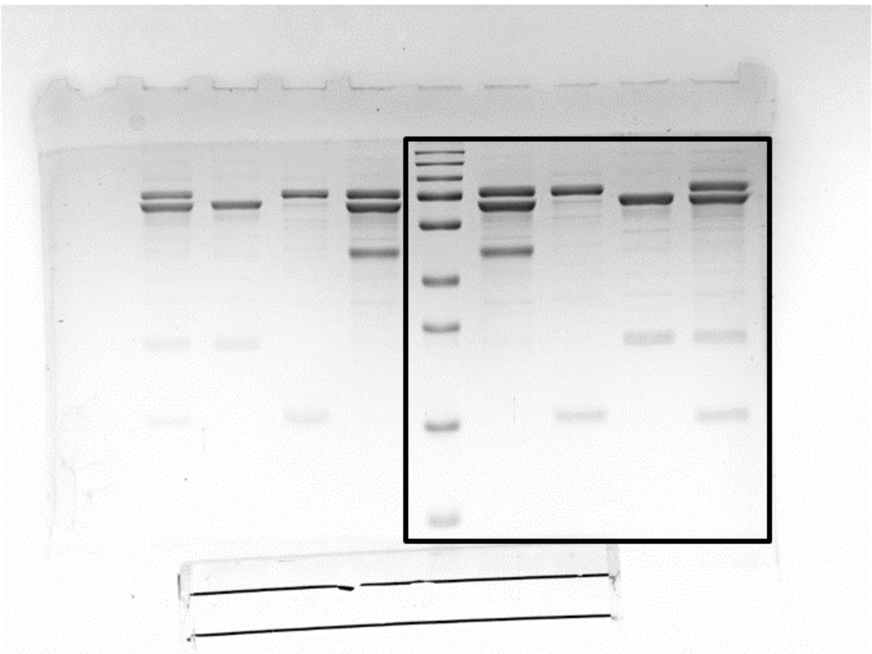

Supplementary Figure 8. Uncropped and unedited SDS-PAGE image for figure 2a. The part in the box was used to show the result.

Supplementary References

1. Bae, S. S. *et al.* *Thermococcus onnurineus* sp, nov., a hyperthermophilic archaeon isolated from a deep-sea hydrothermal vent area at the PACMANUS field. *J. Microbiol. Biotechnol.* **16**, 1826–1831 (2006).
