# Supplementary material for: Bioconversion of CO to formate by artificially designed carbon monoxide:formate oxidoreductase in hyperthermophilic archaea
Source: Commun Biol. 2022 Jun 3;5:539. doi: 10.1038/s42003-022-03513-7 (PMC9166738; doi:10.1038/s42003-022-03513-7)
Supplement: Supplementary file 3 — Description of Additional Supplementary Files [file 42003_2022_3513_MOESM3_ESM.pdf]

Description of Additional Supplementary Files

File name: Supplementary Data 1

Description: Original data for main figures.

File name: Supplementary Data 2

Description: Original data for supplementary figures.
